# Current and Future Advances in Surgical Therapy for Pituitary Adenoma

**DOI:** 10.1210/endrev/bnad014

**Published:** 2023-05-19

**Authors:** Danyal Z Khan, John G Hanrahan, Stephanie E Baldeweg, Neil L Dorward, Danail Stoyanov, Hani J Marcus

**Affiliations:** Department of Neurosurgery, National Hospital for Neurology and Neurosurgery, London WC1N 3BG, UK; Wellcome/EPSRC Centre for Interventional and Surgical Sciences, University College London, London W1W 7TY, UK; Department of Neurosurgery, National Hospital for Neurology and Neurosurgery, London WC1N 3BG, UK; Wellcome/EPSRC Centre for Interventional and Surgical Sciences, University College London, London W1W 7TY, UK; Department of Diabetes & Endocrinology, University College London Hospitals NHS Foundation Trust, London NW1 2BU, UK; Centre for Obesity and Metabolism, Department of Experimental and Translational Medicine, Division of Medicine, University College London, London WC1E 6BT, UK; Department of Neurosurgery, National Hospital for Neurology and Neurosurgery, London WC1N 3BG, UK; Wellcome/EPSRC Centre for Interventional and Surgical Sciences, University College London, London W1W 7TY, UK; Digital Surgery Ltd, Medtronic, London WD18 8WW, UK; Department of Neurosurgery, National Hospital for Neurology and Neurosurgery, London WC1N 3BG, UK; Wellcome/EPSRC Centre for Interventional and Surgical Sciences, University College London, London W1W 7TY, UK

**Keywords:** pituitary adenoma, transsphenoidal, artificial intelligence, robotics, augmented reality, digital health

## Abstract

The vital physiological role of the pituitary gland, alongside its proximity to critical neurovascular structures, means that pituitary adenomas can cause significant morbidity or mortality. While enormous advancements have been made in the surgical care of pituitary adenomas, numerous challenges remain, such as treatment failure and recurrence. To meet these clinical challenges, there has been an enormous expansion of novel medical technologies (eg, endoscopy, advanced imaging, artificial intelligence). These innovations have the potential to benefit each step of the patient’s journey, and ultimately, drive improved outcomes.

Earlier and more accurate diagnosis addresses this in part. Analysis of novel patient data sets, such as automated facial analysis or natural language processing of medical records holds potential in achieving an earlier diagnosis. After diagnosis, treatment decision-making and planning will benefit from radiomics and multimodal machine learning models. Surgical safety and effectiveness will be transformed by smart simulation methods for trainees. Next-generation imaging techniques and augmented reality will enhance surgical planning and intraoperative navigation. Similarly, surgical abilities will be augmented by the future operative armamentarium, including advanced optical devices, smart instruments, and surgical robotics. Intraoperative support to surgical team members will benefit from a data science approach, utilizing machine learning analysis of operative videos to improve patient safety and orientate team members to a common workflow. Postoperatively, neural networks leveraging multimodal datasets will allow early detection of individuals at risk of complications and assist in the prediction of treatment failure, thus supporting patient-specific discharge and monitoring protocols.

While these advancements in pituitary surgery hold promise to enhance the quality of care, clinicians must be the gatekeepers of the translation of such technologies, ensuring systematic assessment of risk and benefit prior to clinical implementation. In doing so, the synergy between these innovations can be leveraged to drive improved outcomes for patients of the future.

## Graphical Abstract

**Graphical abstract:**
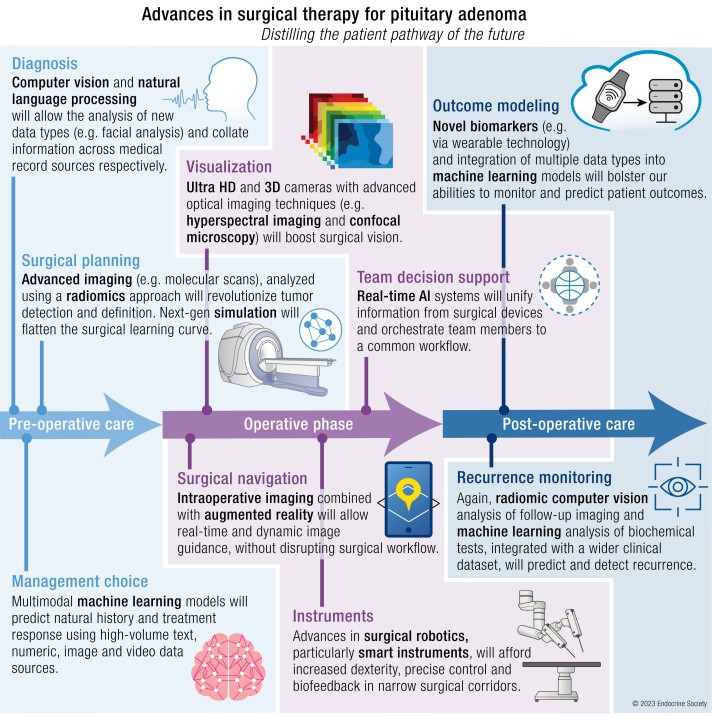

**Essential Points**

- Contemporary challenges and their solutions have been identified and segmented into 3 phases of the pituitary patient pathway: the preoperative, intraoperative, and postoperative phases
- Medical image computing, computer vision, and natural language processing will harness novel data sets to achieve an earlier and more accurate diagnosis
- Decision-making will be enhanced through advanced preoperative imaging and multimodal machine learning models—allowing personalised management strategies, tailored to predicted treatment response
- Surgical safety will be improved by novel intraoperative imaging and augmented reality, providing improved surgical navigation
- The next generation of tools to equip the pituitary surgeon, including advanced optics, surgical robotics, and smart instruments, will maximise safe surgical resection
- A surgical data science approach using real-time AI systems will improve operative workflow, safety, and team performance
- Novel biomarkers, computer vision, and machine learning will provide early-warning systems for complications, identify recurrence, and predict remission—reshaping the postoperative care of this patient group

Pituitary adenomas are among the most common intracranial tumors, with an estimated prevalence of up to 20% (1, 2). They are slow-growing tumors, with numerous subtypes, broadly divided into nonfunctioning adenomas and functioning adenomas (1, 2). They may present incidentally, through mass effect (eg, visual decline), or hormone imbalance (eg, Cushing disease), and can potentially cause significant morbidity, quality-of-life reduction, and death if left untreated (1-3).

Management paradigms for pituitary adenomas have been dynamic, with advances in imaging, hormone therapies and surgical technology impacting guidelines significantly (4-6). Recently, numerous practice variations were adapted in light of the COVID-19 virus, including alterations in interventional procedures, hormonal therapy, and monitoring for safe service delivery to pituitary patients (7, 8). The foundation of this agile and advancing treatment landscape is the collaboration of the multidisciplinary team caring for patients with pituitary adenomas in concert (7, 8). A further example of this is the emergence of Pituitary Centres of Excellence, consolidating the necessary expertise into fewer, but resultantly higher volume, specialist centres—to drive improvement in patient outcomes (9). This is particularly relevant for surgical management of these tumors—which has the potential to offer cure, and thus, is the cornerstone of treatment for the majority of symptomatic pituitary adenomas (9-12). Transsphenoidal surgery is technically demanding with steep learning curves, and thus, service streamlining to maximize surgical team experience and the resulting creation of dedicated subspecialty training programs has helped to improve operative outcomes (9-12).

Despite these organizational and technological improvements in management, many series describe high rates of treatment failure and recurrence—in functioning adenomas (eg, up to 20% in Cushing disease) and nonfunctioning adenomas (eg, up to 50% on long-term follow-up) (13, 14). This is influenced by significant challenges across the patient pathway from diagnosis to follow-up. To meet these clinical challenges, there have been numerous advances in the surgical treatment of pituitary adenomas, with the field benefiting from the recent enormous expansion of novel medical technologies, such as endoscopy, advanced imaging, and artificial intelligence, as well as advances in medical therapies (15, 16). These innovations have the potential to benefit each step of the patient’s journey, and ultimately, drive improved outcomes.

Thus, we aim to explore the scope of existing challenges and potential technological advances in pituitary adenoma surgery from (i) diagnosis and preoperative planning, (ii) surgical proficiency, and (iii) postoperative monitoring—distilling the patient pathway of the future.

## Advances in Preoperative Care

The pituitary adenoma patient pathway starts with a timely and accurate diagnosis, followed by an individualized assessment of suitability for treatment. Despite best efforts, there exist numerous barriers to the multidisciplinary team achieving consistent and universal early diagnosis and treatment. Technological innovations may hold the solution to many of these barriers, and herein we provide examples with potential translational value (Table 1).

**Table 1.** Summary of the contemporary challenges across the pituitary patient pathway with the corresponding current and emerging technological solutions

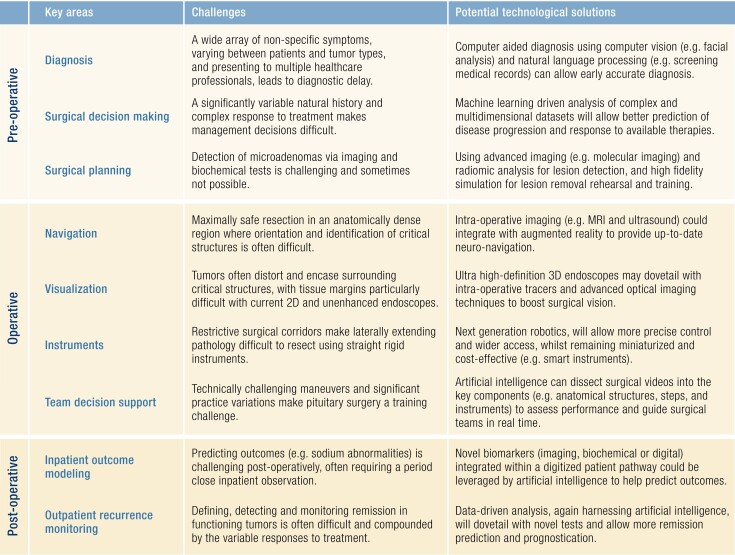

### Diagnosis

#### Challenges

The question of a diagnosis of pituitary adenoma is usually raised by general practitioners, ophthalmologists, neurologists, and endocrinologists at the first line (17). However, the often incidental, insidious, and nonspecific presentation of many pituitary adenomas means that this is often a challenging diagnosis to make (18). Ultimately, diagnosis requires the unification of a wide array of heterogeneous manifestations from various clinicians of differing specialist backgrounds to raise suspicion of the underlying tumor. Thus, diagnostic delay is common, of considerable duration (for example, up to 5 to 10 years in acromegaly), and compounded by socioeconomic and cultural factors (17, 18). During this lag, the tumor grows, making surgical resection more difficult, particularly if there is invasion into the cavernous sinus, while in functioning tumors, systemic complications of hormone imbalance accumulate (19). This in turn can result in irreversible morbidity and socioeconomic decline, further perpetuating issues with healthcare access and diagnostic delay (20). Thus, earlier diagnosis can maximize the chance of cure and reduce the socioeconomic impact, systemic morbidity, and mortality associated with pituitary adenomas.

#### Potential solutions

Computer-aided diagnosis allows high-throughput analysis of large amounts of data (eg, symptoms and signs), detection of otherwise hidden relationships, and is allegedly free of many human cognitive biases (although subject to an alternative set of biases). These systems are particularly useful in identifying subtle deviations from the norm, and in the analysis of image or video data. One example is computer-based facial analysis, which has the potential to detect subtle and slowly evolving changes in facial morphology that would otherwise be missed by patients, families, and clinicians (21-24). Growth hormone–producing functioning adenomas causing acromegaly may be an ideal candidate for its use; facial and acral features are not only the most common symptoms but are typical and tend to manifest early in the disease course (17, 25-27).

Such analysis involves the identification of key facial landmarks, analysis of landmark relationships in space and their changes across time, and association of these changes with disease states (27). The software has displayed accuracies >80% in recognizing patients with acromegaly and control individuals, often exceeding the diagnostic performance of generalist and expert physicians (21, 27-29). Some software particularly performs well in milder forms of the disease, with more subtle facial changes, again outperforming clinicians (21). The principal limitation of facial analysis is the manual landmark and feature extraction, which is labor-intensive and resource-heavy (21). Advances in artificial intelligence, specifically machine learning (ML) and computer vision, have allowed the automation of facial analysis to a granular level (23, 27). Similarly, there have been advances in smartphone technology, with high-quality 2-dimensional (2D) digital cameras now almost ubiquitous. According to a recent Ofcom report, it is estimated that >80% of UK households own a smartphone, with 71% of those in the lowest socioeconomic bracket still owning a smartphone (30). The prevalence of these devices has resulted in a massive and growing volume of facial photographic data. This data, combined with emerging deep learning approaches to image analysis, provides an opportunity to better characterize the dynamic facial phenotype of acromegaly (27). Its applications are widespread, for example, in passport renewal or government identity services, where it could prompt individuals to attend an early medical review based on facial analysis alone. This offers the potential for widespread population screening (eg, via smartphone self-photos), particularly in populations that may have faced disproportionate difficulties in accessing healthcare (eg, ethnic minorities).

Another example of computer-aided diagnosis is the use of natural language processing (NLP), which has the ability to analyze and integrate large volumes of unstructured text data from various data sources, for example, GP records, specialist letters, and recent discharge summaries. Natural language processing has the potential to automatically analyze medical documentation for clusters of features associated with undiagnosed pituitary adenomas, and flag patients for further review and potential earlier diagnosis (31, 32). There is a wide range of accompanying utilities, including economic benefits (eg, reducing the time and resource burden of searching individual medical files) and clinical decision support via predicting clinical outcomes using further integration with ML algorithms (33).

### Surgical Decision-Making

#### Challenge

The natural history of pituitary tumors is considerably variable, even within subtypes. The prediction of the recovery of endocrine and neurological deficits, particularly after the intervention, remains difficult. These factors influence the decision on when or when not to operate, and the optimal timing of this intervention, often requiring discussion at multidisciplinary meetings. This is particularly the case for the growing elderly population, who often have a narrower window for intervention owing to accumulating comorbidities, and are at higher risk for intervention but are similarly higher risk for decompensation if left without treatment (17). Similarly, for medical therapies, for example, dopamine agonists for prolactinomas, identification of those at risk of medication side effects or those who have partial or nonresponse is important for minimizing disease progression and further treatment planning.

#### Potential solutions

Similar to computer-aided diagnosis, the risk modeling and prognostication for the individual patient involves the assimilation of complex multimodal data with a high number of variables (34-36). Machine learning models, particularly neural networks, outperform the traditional statistical methods by leveraging their ability to utilize complex nonlinear relationships between these prediction variables (34-36). There is emerging evidence of the potential benefit and advantage of this technology in the oncology setting—with some ML models being able to perform risk stratification prior to intervention more accurately than risk calculators based on traditional statistical models (37). Similarly, through the integration of multiple data types (eg, histopathological, imaging, and electronic health record notes), ML models have been able to push the boundaries of treatment response prediction, and even discover new features of prognostic significance (38).

Within pituitary adenoma research, numerous models have been developed to predict complications, gross total resection, and postoperative hyponatremia (39-41). ML prediction of resistance to somatostatin analogues in acromegaly holds promise in guiding more personalized treatment regimes, relying on an array of input variables from patient characteristics, imaging findings, biochemistry, and genetic factors (42-45). Similarly, radiomics modeling using magnetic resonance imaging (MRI) has identified biomarkers of nonresponsiveness to dopamine agonists to treat prolactinoma, indicating the potential to determine groups for earlier consideration of surgical resection (46). Furthermore, radiomics have been demonstrated to aid response to radiotherapy, offering novel means of selecting and counseling patients (47).

However, many of these studies have been based on unidimensional text/numeric data only or imaging data only, and the next steps involve the integration of multimodal granular biomarkers into these models. This dataset would ideally be standardized to establish a core set of preoperative (demographics, comorbidities, functional status, visual function, endocrine status, histopathology, imaging), operative, and outcome data. Such standardization has been achieved through Delphi consensus processes and will be important for the pooling of data across centers, thus improving ML model performance and generalizability (35, 48, 49). The curation of high-quality and high-volume clinical datasets (eg, national registries) will build on this, with concurrent optimization of electronic medical record systems for efficient data harvesting (35, 48). Finally, model development and reporting must also be standardized, and guidelines such as the TRIPOD framework (transparent reporting of a multivariable prediction model for individual prognosis or diagnosis) must be used for model reproducibility and interpretability (50). Clinicians must lead this data stewardship, ensuring it is representative of their treating population, so that the resulting models provide an accurate individualized guide to surgical counseling and decision-making (36).

### Surgical Planning

#### Challenge

Preparation for pituitary adenoma surgery involves a decision regarding objectives (eg, total resection, or debulking to decompress surrounding structures), which informs a surgical plan, which must then be executed effectively and safely. In certain cases, surgical planning is particularly challenging; for example, in Cushing disease, the ACTH-producing microadenoma can sometimes be difficult or impossible to visualize preoperatively and intraoperatively (3). Here, our ability to visualize the tumor is central to an effective surgical resection that spares surrounding normal tissues. Despite advances in imaging and the use of auxiliary investigations (eg, petrosal sinus sampling), failure of a planned lesionectomy is not uncommon, and progression to more radical surgery (eg, hemi- or total hypophysectomy) is required, or medical or radiation therapy if this fails. Furthermore, in cases where lesion visualization and generation of an operative plan is more straightforward, building the surgical proficiency to remove the lesion is challenging—owing to the technically demanding, steep learning curve and comparatively low volume nature of this operation (9, 51). For surgeons in training, the pandemic has made the acquisition of the necessary surgical skills particularly challenging (52).

#### Potential solutions

Tumor visualization and the surgical strategy that follows will be revolutionized by advances in imaging technology and our ability to analyze the data that this generates. Next-generation advanced imaging may allow better lesion detection preoperatively. For example, advances in gradient echo sequences and 7-Tesla MRI allow higher resolution imaging, and may highlight otherwise undetectable microadenomas (53, 54). Similarly, molecular imaging techniques have improved lesion detection by leveraging the metabolic properties of these tumors, for example, fluorodeoxyglucose and methionine positron emission tomography imaging for Cushing disease (55-57). The application of ML has demonstrated the ability to augment the data generated by these imaging modalities, using scene reconstruction to generate thinner slices with noise reduction, improving target area resolution (58, 59). Machine learning can also improve our ability to analyze this data, particularly when a data-driven voxel-by-voxel radiomics approach is used. This is a powerful combination of technologies, potentially allowing highly accurate detection of even the most challenging microadenomas, fine delineation of tumor invasiveness, or prediction of intratumoral characteristics, for example, histological subtypes and proliferative index (60-62).

Once the surgical plan is generated, its precise execution, particularly for surgeons in training, is a formidable beast. Surgical simulation may be an answer to this problem. The spectrum of simulators available for pituitary surgery is wide, from low-fidelity physical simulators using bell-peppers, to high-fidelity simulators utilizing 3-dimensional (3D)-printed advanced materials, sometimes to patient-specific design (63, 64). Virtual and augmented reality platforms often require less surgical equipment, can be dynamic (ie, incorporate fluid pulsations), and have been generated at a patient-specific level, but are limited by their general lack of sufficient haptic feedback (65, 66). Next-generation models will combine advanced materials more representative of human tissue with augmented reality and artificial intelligence for smart simulation—which track and react to surgical actions (eg, bleed or leak cerebrospinal fluid), and automatically assess surgical skills.

## Improving Operative Efficiency, Effectiveness, and Safety

After work-up, a decision for operative management and the careful planning of tumor resection; comes the execution of the operation. The operating *theater* is aptly named, representing the coordinated *performance* by surgeons (often from multiple specialties), anesthetists, and nurses to achieve a singular goal—an efficient, effective, and safe operation. The Royal College of Surgeons *Future of Surgery* report highlights the technologies likely to be most impactful—advanced endoscopes, robotics, augmented reality, virtual reality, and artificial intelligence—integrated together, as we move into the era of “smart” operating theaters (67). Pituitary surgery is no exception, and there are numerous unmet clinical needs that may benefit from these innovations. It is worth noting that most introduction of technology is not systemically assessed, this stands true for many technologies used in endoscopic endonasal surgery (68). The IDEAL (Idea, Development, Exploration, Assessment and Long-term follow-up) framework provides a structured pathway to guide the proportionate evaluation of medical devices (based on their risk profile) and safe stepwise clinical assessment of benefit (69-71). Pituitary adenoma surgery has potentially serious complications, and the introduction of any technology must be carefully assessed using such a framework and encompass operating team human factors (69-71).

### Navigation

#### Challenges

Pituitary adenomas are located in an anatomically rich area, with life-sustaining vessels (eg, carotids) and other critical structures (eg, optic nerves) located within a densely packed region. This anatomy is distorted and sometimes encased by tumors. Intraoperative navigation helps to guide surgeons as to where the tumor and these structures are. This is most commonly done using image-guided systems that require specialized scans and preoperative registration. They provide guidance through the placement of a probe in the field and cross-referencing the position of this probe with its predicted position on the preoperative imaging. While this technology has revolutionized neurosurgery, including pituitary surgery, particularly during challenging/non-standard cases, it has numerous issues. These include interruption to the surgical workflow, for example, the need for registration preoperatively and for intraoperative pauses to use the navigation probe. Additionally, the relative inaccuracy after structures shift intraoperatively (eg, after tumor debulking) limits the utility of the navigation as the operation progresses.

#### Potential solutions

Real-time navigation, that is, a system that provides navigation data that is representative of the surgical field at that moment in time, has been explored using various technologies. Intraoperative MRI is the most studied modality and integrates with existing image guidance systems to update the imaging on which it is based, so that intraoperative tissue shifts are accounted for. Newer high-field MR systems are proposed to particularly highlight the “resectable residuum”—tumor remnants that are safely removable, without a high risk of damage to surrounding neurovascular structures (72). Numerous studies suggest it resultantly improves the extent of resection and assists in the assessment of neurovascular decompression, for example, chiasmal decompression in those with visual loss (73-75). Similarly, it provides immediate feedback and quality control to surgeons, which may have benefits in training and flattening of operative learning curves (72, 76). However, intraoperative MRI is resource-heavy, requiring changes to most of the operating room infrastructure, for example, magnetic shielding and acquiring MR-compatible equipment (72). Furthermore, it significantly interrupts operative workflow, which has to cease for imaging to take place and thus prolongs both surgical and anesthetic time (72, 77).

Intraoperative ultrasound addresses some of the disadvantages of intraoperative MRI—being less disruptive to workflow, less time-consuming, and significantly cheaper. Unlike intraoperative micro-Doppler (used for internal carotid artery identification), it seeks to assist with tumor identification (eg, Cushing disease microadenoma) and delineation of the tumor-gland interface (78). Initial issues highlighted with this technology included large probe size, image resolution quality, and operator dependency. Recent improvements in probe miniaturization and image quality have made this technology a candidate for translation, with first-in-human studies (IDEAL Stage 1) suggesting the feasibility and safety of this device (79).

Synergy with augmented reality platforms is proposed to improve the efficiency of these navigation systems even further, allowing the integration of information from imaging modalities such as MRI onto surgical display fields (eg, endoscopic video) via overlay (80-82). These systems do not require probes, or extra monitors, and build 3D models directly onto the surgical field for more intuitive navigation with improved 3D perception and minimal disruption to operative workflow (80-82). Studies suggest this may help achieve more tumor resection with less collateral neurovascular damage, particularly in revision cases with distorted anatomical landmarks (80-82). For this augmented reality to be real-time, ie, accounting for intraoperative tissue shifts, then up-to-date information must be fed into the system via intraoperative imaging as above, or alternatively, through a combination of preoperative imaging and computer vision–based analysis of intraoperative video (eg, to detect intraoperative anatomy and events), which is discussed in detail later.

### Visualization

#### Challenges

Pituitary tumors, housed in an anatomically complex region of the skull base, at the end of a long and narrow surgical corridor, command rich visualization during attempts at surgical resection. This is compounded by the fact that many tumors can distort this anatomy, and be composed of various consistencies and subcomponents, making distinguishment of tumor margins and extent difficult. Additionally, many tumors can be too small to distinguish macroscopically from normal tissue (72). It is no surprise that the advent of endoscopy is regarded by many as the greatest technological advance in modern pituitary surgery, boosting a surgeon's visualization intraoperatively, with a wider and more illuminated field of view. However, most endoscopes are 2D, requiring depth perception estimation by surgeons through anatomical and motion cues. Similarly, the tumor-normal tissue interface is often challenging, particularly for microadenomas, invasive tumors, and revision surgeries.

#### Solutions

Augmentation of surgical visualization technology is a rapidly expanding space, with improvements in image quality, ergonomics, and synergy with complementary technologies among the principal drivers for this expansion. High definition technology (including 4K Ultra HD) affords state-of-the-art image resolution, and in the context of pituitary surgery, allows better discrimination of tumor and gland with a potential for reducing unexpected tumor residuals (when compared to standard definition cameras) (83, 84). Similarly, 3D endoscopes seek to improve the appreciation of depth through the added shape and contour information provided to surgeons. While in many endoscopes this is simulated digital depth perception rather than the binocular stereopsis of the microscope, numerous studies support its utility in complex or extended endonasal procedures, although there are notable issues such as motion sickness for some users and potential disruption to workflow due to the need for increased intraoperative cleaning of the endoscope (eg, nasal mucosa bleeding may block one of the two cameras within the endoscope required for 3D vision) (85, 86). However, the translation of these intraoperative benefits into postoperative outcomes, when compared with 2D endoscopy, is less well established and calls for further systematic, structured assessment (ie, via the IDEAL pathway) (70, 87).

Nevertheless, these advances have the potential for synergy with complementary innovations. For example, 3D endoscopy may provide a richer foundation for a more detailed augmented reality overlay in the future. Similarly, high-definition scopes may potentiate the benefits of intraoperative tracers and dyes. Numerous chemicals have been tested, such as 5-ALA (no demonstrated benefit in pituitary adenoma tumor identification), ICG (may help in identifying functional adenomas and internal carotid arteries), OTL38 with near-infrared imaging (may help in identifying nonfunctioning adenomas with high folate receptor expression), and fluorescein (may help in identifying functional adenomas) (88-90) Innovation in advanced optical imaging is particularly exciting and builds on the use of these tracers and dyes. For example, probe-based confocal endomicroscopy, allowing granular tissue characterization based on microstructural features, can be used with fluorescein to obtain digital diagnostic biopsies of pituitary tumors (91-93). Similarly, hyperspectral imaging leverages the ability to analyze the chemical composition of tissue, allowing more precise tumor delineation (72, 93, 94).

Recently, there has been increasing awareness of the need to incorporate surgical ergonomics into device development (70, 95). One example is the use of exoscopes, which when compared to microscopes, allow a more comfortable posture during surgery, with a smaller operating room footprint, and the potential for integration with concurrent endoscope use via a split screen. However, concerns with the resolution (when compared with a microscope) and the width of visualization (when compared with the endoscope) have hampered their routine uptake (96, 97) Furthermore, ergonomics-orientated robotic devices, such as endoscope holders and surgical armrests (for the endoscope holding arm), have been developed to reduce surgeon fatigue and stabilize the surgeon's hand during pituitary surgery (98). Similarly, robotic endoscopes with adjustable viewing angles (15-90 degrees) have the potential to allow wider visualization without the need for switching between multiple scopes (99).

### Instruments

#### Challenges

The narrow nasal surgical corridor which has challenged visualization also tests the capabilities of contemporary surgical instruments. Limitations imposed by this restrictive space and the fulcrum effect results in restricted instrument reach and co-axial movement of the instruments with challenging surgical triangulation (95). This not only contributes to the steep learning curve of pituitary surgery but also makes invasive tumors, for example, those extending into the cavernous sinus, very difficult to access. More generally, the forces used in neurosurgery, including pituitary tumor resection, are among the lowest of all surgical specialties (100). Thus, not only must these surgical tools be small enough the pass through the nasal passage and dexterous enough to provide bimanual control, but they must also be particularly precise with sensitive haptic feedback, so that tool-tissue forces are carefully controlled (95).

#### Potential solutions

Recent advances in engineering and materials have allowed miniaturization while retaining precise kinematic control, careful force control, and haptic feedback in surgical robotics. These advances will herald a new era of devices capable of meeting the needs of neurosurgical procedures. Surgical robotics can be categorized into supervisory controlled (pre-programmed to carry out a specific task), telesurgical (surgeon remotely controls the robot in real time), and shared-control (surgeon physically controls the robot in real time). The most successful robotic system, the Da Vinci (Intuitive Surgical) is a telesurgical system, and despite efforts to miniaturize the system, the endonasal approach presents too narrow of a corridor for its use, although some surgeons have used the system transorally (101). Numerous other telesurgical systems are in development but only preclinically. For example, systems with flexible tubular shafts which fit within the nose and move using tendon pulley systems with concentric tubes, contorting the tubular shaft and bringing the end-effector (ie, grasper) to the surgical target with 6 degrees of freedom (102). Flexible robots are the cornerstone of soft robotics, a subfield which uses bio-inspired design and nonrigid materials to create systems which are more maneuverable (eg, snake-like) and less damaging to surrounding tissue (103). Conceptually, these devices are well suited to the delicate nature of neurosurgery, but issues with the controllability and sterilizability of current technology are barriers to development and adoption (103).

More recently, there has been an explosion in the development of “smart instruments” (ie, shared-control robotic systems) that are wielded by the surgeon to augment their own abilities (95). One example is the use of articulated instruments which increase surgical access beyond the straight axes of the nasal corridor, with joystick-like control of the end-effector (104, 105). Preclinical (IDEAL Stage 0) validation of these instruments is promising, outperforming standard rigid surgical instruments in terms of dexterity, control, and ergonomics, while having the added ability to gather important surgical data through sensors (eg, force applied) which could be feedback to surgeons in real time (106, 107).

Ultimately, whether these instruments are rigid or soft, telesurgical, or shared-control, as invasive and potentially high-risk devices they must undergo proportionate rigorous and systematic assessment for effectiveness, safety, and cost-benefit prior to integration into operating theaters of the future (70).

### Team Decision Support

#### Challenges

Pituitary surgery is technically challenging, and has steep learning curves, with practice variations across centers and countries (11, 108-110). This leads to varying surgical outcomes along the learning curve and from center to center. This presents significant training challenges and raises the question as to which aspects of practice (ie, surgical steps) are optimal and how best to learn them. However, no two surgeries are the same, and therefore interrogating differences in the performance of surgeries and generating comparative evidence between surgical techniques and technologies is challenging. Intraoperative decisions are therefore often via expert apprenticeship or reactively via trial and error. Historically, the resources required to extract the necessary data from surgical procedures to a granular level, and the number of variables and volume of data needed for meaningful analysis, meant answering these training and practice challenges was almost totally infeasible.

#### Potential solutions

The first step to answering many training and practice challenges in pituitary surgery and providing guidance to surgeons of the future is surgical workflow analysis (108). This involves systematically breaking down operations into key phases and steps, codifying surgery into its fundamental building blocks. There is international consensus on the key phases and steps of pituitary surgery, but analyzing surgeries in this fashion, for example, via review of operative videos, is very time and labor-intensive when done manually (108, 111, 112). By applying ML and computer vision to operative videos, we can perform this workflow analysis automatically and accurately (111-113).

This AI-driven analysis has numerous potential benefits. First, it generates a library of annotated videos and performance metrics (eg, step duration and order), which can be reviewed by trainees and used for individualized coaching on surgical technique (ie, directing training to particular steps of concern) (113, 114). Secondly, this technology can be used in real time and presented to the surgical team using intraoperative displays with the AI predicting current and future steps. This may improve operational efficiency during surgery, orchestrating the entire team to a common workflow, for example, highlighting the instruments needed next to the scrub technician or upcoming critical steps to the anesthetists (113).

Furthermore, this technology provides the foundation for numerous avenues of further analysis. For example, computer vision–based detection of anatomical structures (eg, optic nerves or carotid arteries) is triangulated to particular surgical steps, such as high-risk steps during tumor resection where the risk of neurovascular injury is highest. This information can again be used for educational retrospective review for trainees or in real time, to guide surgeons intraoperatively. Through recognition of the normal pituitary gland, delineating tumor margins may be easier. Similarly, the recognition and tracking of surgical instrument use and movement across critical operative steps may provide useful feedback for surgical trainees on their economy of movement and optimal kinematics (115). This data could be integrated with “smart” instrument force data and anatomical data (using videos and navigation technology) and displayed using augmented reality to guide surgeons on the optimum maneuvers (instrument use), at the optimum time (step) and place (anatomy). Future operating theaters will host these technologies and other innovations (eg, wearable cardiorespiratory and neurosensory monitoring for staff) in concert, connecting them and all members of the operative team. If and when these smart theaters are widespread, and our performance is linked to postoperative outcomes, this technology may go further than simply orientating the team and may provide outcome-driven guidance to surgeons in real time—heralding the era of truly “information-guided” surgery (67, 116).

## Optimizing Postoperative Care

Once the surgical challenge of resecting the pituitary lesion has been surmounted, the postoperative phase commences. Postoperative care can be divided into inpatient and outpatient stages, which have distinct challenges. The inpatient phase involves recovery from surgery, monitoring for surgical complications and initial outcomes. In the outpatient phase, the suspected diagnosis is confirmed, and surveillance begins. Both look to stratify patients by risk; however, achieving such foresight consistently remains a challenge.

### Inpatient Outcome Modeling

#### Challenges

Predicting outcomes is notoriously difficult after pituitary surgery, including for the most common complications, such as sodium abnormalities and cerebrospinal fluid rhinorrhea (109, 117-119). This results in the need for extended monitoring of patients postoperatively, and some groups have trialed prophylactic therapies on a blanket basis to prevent these common complications, for example, fluid restriction for syndrome of inappropriate antidiuretic hormone (SIADH) or bed rest for cerebrospinal fluid rhinorrhea (120). The core issue is our ability to accurately predict, and risk stratify patients postoperatively.

#### Potential solutions

Traditional methods have likely failed due to the need for multimodal datasets, containing a large number of variables with complex nonlinear relationships to answer this particular unmet need. However, ML tools, especially neural networks, have the ability to analyze these datasets (36). For example, intraoperative workflow analysis can be integrated into multimodal AI models with preoperative and postoperative data, such that the patients can be classified into high- and low-risk groups for each surgical complication (121). High-risk groups may benefit from extended monitoring with closer attention to potential complications or prophylactic treatments, while low-risk groups may benefit from early discharge and fast-track protocols (sparing risks of nosocomial disease and streamlining resource allocation) (117, 122).

Furthermore, the development of novel biomarkers may supplement the above datasets or stand as independent predictors for patient outcomes. Many of these biomarkers have been diagnosis-orientated, and there is a growing appreciation for the clinical need for these biomarkers in the postoperative care phase. For example, novel imaging techniques such as optical coherence tomography angiography provide a rapid noninvasive assessment of retinal microvasculature changes and may predict those who have structural retina improvements and functional vision recovery after surgery (123). Similarly, digital biomarkers may be generated using active self-reporting of symptoms by patients via smartphone applications (122, 124). When combined with a validated set of patient-reported outcome measures, which has recently been developed for patients undergoing pituitary surgery, this may generate a digital dataset otherwise unrepresented in traditional outcome reporting (125). However, as the age of big data continues its growth, careful interrogation of the bias within the data-driven analysis is paramount. If a subset of patients (eg, those with severe visual or functional disability) are unable to access and contribute to these biomarker datasets, resulting predictive models will not be valid in these populations. In the era of innovation, basic principles stand true, and the multidisciplinary pituitary team must ensure that translated technologies are fair, equitable, and accessible to the patients they care for.

### Outpatient Recurrence Monitoring

#### Challenges

For patients, clinicians, and health systems, remission is an important treatment goal. It is challenging to define in functioning tumors, owing to the limitations of present methods of defining remission and the variances in individual responses to surgical and adjuvant treatment (108, 126-128). Deciding upon remission is fundamental for Cushing disease, as it aids neurosurgical decision-making with regard to more aggressive surgical resection of suspected lesions, hemi-gland, or even total removal of the pituitary gland (129-131). In acromegaly, reliance on medication postoperatively leaves the patient vulnerable to treatment resistance. From a systems perspective, medical management of acromegaly is costly; thus, remission provides gains for the wider health system, alongside the many individual benefits to the patient (132).

#### Potential solutions

Again, a data-driven machine learning approach has shown promise in outpatient surveillance; for example, it has been shown to outperform present prognostic biomarkers in determining remission in acromegaly, computing arrays of established variables in new ways to predict outcomes (42, 133, 134). Single-center studies show promise in determining surgical success and endocrine outcomes, offering tailored treatment and follow-up approaches according to the likelihood of remission. Identifying treatment failures sooner will support definitive treatment decision-making, showing value in producing reliable and accurate prediction models of remission. Early identification of remission supports earlier discharge and outpatient monitoring. Preoperative, intraoperative, and day 1 postoperative variables have been used to model early remission, outperforming established prognostic factors. Similarly, prognostic factors in Cushing disease have been identified to associate with recurrence or remission (135-138). Preoperative variables can be used to estimate immediate remission, supporting enhanced recovery pathways and reductions in length of stay (117, 139). In patients with delayed remission, decision-making remains a challenge, considering the outcome uncertainty and urge to achieve remission, placing value on prediction models identifying this subgroup of patients (140). More generally, risk stratification can aid medical or radiotherapeutic adjuncts with earlier consultation of endocrinologists or oncologists in patients expected to respond poorly to surgery. Accurate prediction of remission could influence established treatment paradigms. First-line surgery for prolactinomas remains controversial, as medical therapies are easily available; however, means of predicting surgical success and remission, coupled with increasing surgical safety may become more accepted as a treatment option (141).

## Conclusions

We have the potential to significantly improve the lives of patients with pituitary adenomas due to recent advances in surgical, medical, and radiological therapies. However, surgical treatment failure is still a common problem and is influenced by significant challenges across the patient pathway—including diagnosis, preoperative planning, surgical proficiency, and postoperative care. The patient pathway of the future will integrate novel surgical technologies—working in synergy with each other and in harmony with the multidisciplinary team. Clinicians must be the gatekeepers of technological translation, ensuring systematic assessment of risk and benefit, and leveraging these innovations to drive improved outcomes for patients of the future.

## Abbreviations

2D: two-dimensional
3D: three-dimensional
AI: artificial intelligence
IDEAL: Idea, Development, Exploration, Assessment, Long-term study
ML: machine learning
MRI: magnetic resonance imaging
